# Trial of labour after two caesarean sections (TOLA2C) and risk of uterine rupture, a retrospective single centre study

**DOI:** 10.1186/s12884-024-06763-x

**Published:** 2024-09-03

**Authors:** Sonja Denjean, Daniela Reis, Miriam Bräuer, Alfred Längler

**Affiliations:** 1 Department of Obstetrics, Gemeinschaftskrankenhaus Herdecke, Herdecke, Germany; 2 Department of Pediatrics, Gemeinschaftskrankenhaus Herdecke, Herdecke, Germany; 3 Professorship for integrative pediatrics, Institute for integrative Medicine, Witten/Herdecke University, Witten, Germany; 4 Gynaecology and Obstetrics, Diakonie Klinikum Schwäbisch Hall, Schwäbisch Hall, Germany

**Keywords:** Trial of labour after two caesarean sections (TOLA2C), Uterine ruptures, Elective repeat caesarean section (ERCS), VBAC-2

## Abstract

**Background:**

Most German hospitals do not offer a trial of labour after two caesarean sections (TOLA2C). TOLA2C is claimed to be associated with too many complications, above all the high risk of uterine rupture. The objective of this study is to review our experience with TOLA2C, with special attention paid to the risk and probability of uterine ruptures. Secondary outcomes include comparing neonatal and maternal outcomes in the group of TOLA2C with the group of elective repeat caesarean section (ERCS) and to assess the success rate for vaginal birth after two caesarean sections (VBAC-2).

**Methods:**

The retrospective cohort study was conducted in a community hospital in North Rhine-Westphalia. Inclusion criteria were all pregnant women with two caesarean sections in their medical history, with a current vertex singleton pregnancy and the absence of morphological abnormalities of the foetus, who gave birth in our facility between January 2015 and June 2021. Descriptive statistics were calculated and Kolmogorov-Smirnov tests, Mann-Whitney U tests, Fishers exact tests, Chi^2^ –tests and t-tests for independent samples were performed.

**Results:**

A total of 91 cases were included in the TOLA2C-group. These were compared to 99 cases that, within the same time frame, had an elective repeat caesarean section (ERCS-group). There was no statistically significant difference found in the neonatal outcome between the two groups (except for the neonatal pH-value: p 0.024). The hospital stay was significantly shorter in the TOLA2C-group, while maternal complication rates were almost similar (13.2% in the TOLA2C-Group, vs. 16.2% in the ERCS-Group). The success rate for TOLA2C was 55%. No complete uterine rupture was found, but in three cases an incomplete rupture (3.3% rate for incomplete uterine ruptures) occurred, but had no influence on the neonatal outcome.

**Conclusion:**

TOLA2C is not associated with a worse maternal or neonatal outcome compared to ERCS, and especially the risk of complete uterine ruptures seems to be low. TOLA2C should be more widely offered to suitable patients who are motivated for it.

## Introduction

Over the past several decades, caesarean section rates have steadily increased worldwide [1, 2] leading to an increasing number of births after one or more caesarean sections (c-sections), with a corresponding increase in maternal and infant morbidity [3–5]. This, in turn, affects health care systems [6, 7]. In Germany, as in most countries around the world, it is common practice to recommend, if possible, a vaginal delivery in the pregnancy after a c-section. The benefits of vaginal birth after one c-section have been proven in various studies [8–11]. After two c-sections, it has become common practice in Germany, as well as in many other countries, to recommend an elective repeat caesarean section (ERCS) as the mode of delivery for a subsequent pregnancy. In most hospitals in Germany, a trial of labour after two caesareans (TOLA2C) is not possible. This is justified to patients by stating low success rates, an increased number of complications [12] and especially the potential risk of uterine rupture [10, 13]. Although recent publications [14–18] have shown that TOLA2C does not seem to be associated with an increased risk for mother and child, TOLA2C is still a rarity in Germany.

In the Department of Obstetrics at the Gemeinschaftskrankenhaus Herdecke, it has been possible for many years to attempt a vaginal birth after multiple c-sections, if conditions and risk factors are carefully evaluated and patients counselled appropriately. The aim of this study is to investigate the risk of uterine rupture in TOLA2C and to compare the maternal and foetal outcome of TOLA2C to ERCS. Furthermore, the success rate of TOLA2C as well as the risk of emergency c-section under general anaesthesia in TOLA2C will be analysed. We also tried to identify factors associated to a successful or a failed TOLA2C.

## Methods

The retrospective cohort study was conducted in the Gemeinschaftskrankenhaus Herdecke, a community hospital in North Rhine-Westphalia from January 1, 2015 to June 30, 2021. The Gemeinschaftskrankenhaus Herdecke is a community teaching hospital with a perinatal centre and approximately 2.000 deliveries per year. The study was conducted according to the Declaration of Helsinki [19] and was reported according to the STROBE guidelines for reporting observational cohort studies [20].

The cases were identified by manually looking through the birth registers, and verifying possible cases within the digital patient files, with the hospital documentation system (ORBIS), as there is no International Classification of Disease Code for women with a history of two prior caesareans in Germany. Inclusion criteria were all pregnant women with two c-sections in their medical history, with a current vertex singleton pregnancy and the absence of morphological abnormalities of the foetus. All included women had only low transverse uterine incisions in their previous caesareans, as this is a requirement for the possibility to undergo TOLA2C in the Gemeinschaftskrankenhaus Herdecke.

Uterine ruptures were defined as either a complete uterine rupture (complete division of all three layers of the uterus) or an incomplete uterine rupture (the peritoneum overlying the uterus is intact, the uterine contents remain within the uterus). Asymptomatic uterine scar ruptures were reported as well.

The collected data included demographic data such as age, gravidity, parity, as well as medical data including comorbidities, indications of previous caesareans, maternal complications, hospital stay, induction of labour, anaesthesia during the birth (local, epidural, spinal, general anaesthesia), application of oxytocin during a TOLA2C. Postpartum haemorrhage was defined as a blood loss of more than 1.000 ml during delivery. Neonatal parameters reported included APGAR, pH-Value at birth, birthweight, neonatal intensive care unit (NICU) admissions and duration of the hospital stay at NICU.

The statistical analysis was performed with SPSS (Version 24.0. SPSS Inc., USA), using the Kolmogorov-Smirnov tests, Mann-Whitney U tests, Fishers exact tests, Chi^2^ –tests and t-tests, as all but one of the relevant variables were not normally distributed according to the Kolmogorov-Smirnov test.

## Results

A total of one hundred and ninety women were included in our analysis. There were 91 women within the TOLA2C-Group, which were compared to the 99 cases of the ERCS-Group (see Table 1).

**Table 1:** Sociodemographic and medical data of TOLA2C and ERCS

	TOLA2C	ERCS	*p*-values
Study participants	*N* = 91	*N* = 99	
Age mother median (min., max.)	35 (21, 44)	35 (21, 44)	*p* = .309^a^
Gravidity median (min., max.)	4 (3, 9)	4 (3, 10)	*p* = .380^a^
Parity median (min., max.)	3 (3,7)	3 (3,7)	*p* = .961^a^
Gestational age (week) mean (SD)	39.90 (1.24)	38.87 (1.09)	*p* < .001^b**^
Prior vaginal delivery	*n* = 17 (18.7%)	*n* = 16 (16.2%)	*p* = .520^c^
**Maternal Outcome**			
Complications	*n* = 12 (13.2%)	*n* = 16 (16.2%)	*p* = .683^d^
Hospital stay (days) mean (SD)	2.74 (1.39)	3.22 (1.11)	*p* < .003^a**^
**Neonatal Outcome**			
APGAR 5 min median (min., max.)	10 (6, 10)	10 (6, 10)	*p* = .234^a^
APGAR 10 min median (min., max.)	10 (7, 10)	10 (7, 10)	*p* = .355^a^
pH-value median (min., max.)	7.30 (7.05, 7.41)	7.33 (7.06, 7.48)	*p* = .024^a*^
Duration of stay in NICU (days) median (min., max.)	5 (2, 7)	5 (3, 19)	*p* = .860^a^
NICU Admission	*n* = 7 (7.7% )	*n* = 8 (8.1%)	*p* = 1.000^d^
Risk for emergency c-section in general anaesthesia	1.1% (*n* = 1)	1,0% (*n* = 1)	

^a^Mann-Whitney U test; ^b^ t-test; ^c^ Chi^2^ –test Pearson Chi-Square; ^d^ Fishers exact test

Abbreviations: TOLA2C, trial of labour after two caesareans; ERCS, Elective repeat caesarean section; NICU, Neonatal intensive care unit; c-section, caesarean section

^**^p < .01; ^*^p < .05

Within the TOLA2C-Group 35% (*n* = 32) of the women had a spontaneous delivery and 19.8% (*n* = 18) of the women had an assisted vaginal delivery (mainly vacuum extraction), while in 45% (*n* = 41) of the cases VBA2C was not possible and a c-section was performed. The success rate for a vaginal delivery was 55%.

The percentage of women, who have had a previous vaginal delivery, was almost similar between the two groups, showing that not only women who had already delivered spontaneously were encouraged to go for a TOLA2C in our collective (see Table 1).

The maternal outcome was slightly better in the TOLA2C-group than in the ERCS-group due to a significantly shorter hospital stay (see Table 1), while complications were almost similar between the two groups. The most common complication in both groups was postpartum haemorrhage.

Neonatal outcome was similar between the two groups (apart from the pH-value, which has no clinical consequences), as well as the risk for an emergency caesarean in general anaesthesia (see Table 1). No maternal or neonatal death occurred.

**Fig. 1:**
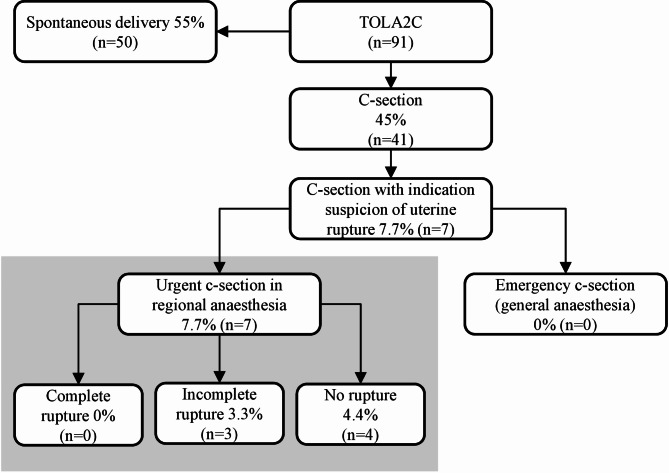
TOLA2C and risk of uterine rupture. Legend: Abbreviations: TOLA2C, Trial of labour after two caesarean sections; c-section, caesarean section

There was no complete uterine rupture in the TOLA2C-group, but three incomplete uterine ruptures were found (risk for incomplete uterine rupture 3.3% within TOLA2C-group) (see Fig. 1), which had no influence on the neonatal outcome (Table 1). Suspicion of uterine rupture was the fourth common indication for a caesarean in the TOLA2C-group (after arrest of dilation, pathological CTG and failure of induction of labour). Five asymptomatic uterine scar ruptures (four in the ERCS group, one in the TOLA2C group) were reported, which did not affect the maternal or neonatal outcome.

Maternal obesity (BMI > 30 at the beginning of pregnancy) (*p* < .027) and a higher neonatal weight at birth (*p* < .019) were factors associated with a failed TOLA2C (see Table 2). Induction of labour, augmentation of labour or epidural anaesthesia did not influence the success or the failure of VBA2C.

**Table 2:** Successful TOLA2C versus failed TOLA2C

	Successful TOLA2C	Failed TOLA2C	*p*-values
Study participants	*n* = 50	*n* = 41	
Age mother median (min., max.)	35 (21, 44)	34 (26, 44)	*p = .*394^a^
Gestational age (week) mean (SD)	39.68 (1.20)	40.17 (1.25)	*p* =.06^b^
Spacing to last c-section in month median (min., max.)	44 (17, 276)	37 (12, 216)	*p = .*652^a^
Birthweight in gram median (min., max.)	3535.00 (2680.00, 4480.00)	3680.00 (2680.00, 4650.00)	*p* = .019^a*^
APGARS @ 5 min median (min., max.)	10 (6, 10)	10 (7, 10)	*p* = .248^a^
APGARS @ 10 min median (min., max.)	10 (7, 10)	10 (9, 10)	*p* = .785^a^
Umbilical cord pH median (min., max.)	7.27 (7.09, 7.41)	7.33 (7.05, 7.41)	*p* < .001^a**^
Hospital stay in days median (min., max.)	2 (0, 5)	3 (2, 9)	*p* < .001^a**^
Prior vaginal delivery (n (%))	12 (24.0%)	5 (12.2%)	*p* = .183^c^
NICU Admission (n (%))	2 (4.0%)	5 (12.2%)	*p* = .237^c^
Induction of labour (n (%))	17 (34.0%)	17 (41.5%)	*p* = .522^c^
Augmentation of labour (Oxytocin) (n (%))	14 (28.0%)	10 (24.4%)	*p* = .636^c^
Epidural anaesthesia (n (%))	24 (48.0%)	20 (48.8%)	*p* = 1.000^c^
Adiposity (n (%))	7 (14.0%)	14 (34.1%)	*p* = .027^c*^
Diabetes (Type I/II, gestational diabetes) (n (%))	7 (14.0%)	7 (17.1%)	*p* = .776^c^

^a^Mann-Whitney U test; ^b^ t-test, ^c^ Fishers exact test

Abbreviations: C-section, caesarean section; NICU, Neonatal intensive care unit;

^**^p < .01; ^*^p < .05

## Discussion

The results of our study show that TOLA2C is a veritable possibility of giving birth for suitable and motivated women, with two previous caesareans in their medical history. TOLA2C is not associated with a worse maternal or neonatal outcome compared to ERCS, and especially the risk of complete uterine ruptures seems to be low. In our collective there was no complete uterine rupture found and the three incomplete ruptures did not affect the neonatal outcome. Recent previous literature finds a complete uterine rupture rate of 0.14–1.6% [14, 15, 18], which confirms our own findings, as well as the prospective multicentre observational study from Landon et al. from 2006 [21].

Our TOLA2C success rate of 55% is lower than in many other publications [13, 15–18, 22], but consistent with a recent population-based cohort study from the US [14]. Our success rate seems realistic, as in our study not mainly women, who have had a previous vaginal delivery, were encouraged to opt for a TOLA2C.

Most patients opting for TOLA2C in Germany are very afraid to have an emergency c-section under general anaesthesia (in Germany, only c-sections under general anaesthesia, with a maximum time frame of 20 min from the decision to perform a c-section to the delivery of the child are called emergency c-sections). In contrast to other publications before [13, 15] we investigated to find out about the risk for emergency c-section under general anaesthesia and found a rate of 1,1% in the TOLA2C group (one case of umbilical cord prolapse after rupture of membranes). As there was one emergency c-section in the ERCS-group too, it seems women opting for TOLA2C do not have a higher risk of an emergency caesarean under general anaesthesia than women opting for ERCS.

As in other recent publications [16, 17] we could show that the hospital stay is significantly shorter in women who opt for TOLA2C than in women who go for ERCS (*p* < .003). It’s even shorter of course, when women have a successful TOLA2C. This circumstance could be an economic inducement to offer TOLA2C more widely.

In our collective of the TOLA2C-group the rate for vaginal assisted births was surprisingly high at 19.8%. Whether this high number is a specific phenomenon of obstetrics in Germany in general, or only specific for the Gemeinschaftskrankenhaus Herdecke, remains unclear, as this was a single-centre study. Further investigations need to be undertaken and women opting for TOLA2C in Germany should be counselled on the elevated risk for a vaginal assisted delivery.

The fact that this study is a single centre study, as well as the retrospective study design are among the limitations of our study. It is a limitation as well that our study (as well as all other publications before this) only analyses the maternal and foetal short-term outcomes, directly associated to the delivery. It would be of great interest to investigate the long-term consequences, benefits, complications and health conditions of mothers and children who opted for TOLA2C compared to those having chosen ERCS.

To increase the acceptance and safety of TOLA2C in Germany and around the world, more publications are needed. We hope that our study can contribute to making TOLA2C more popular in Germany.

## Conclusion

Suitable and motivated women with two caesareans in their medical history should be able to make their own choice for their preferred mode of delivery after being counselled appropriately. According to the current status of research, especially women who plan to have more than three children and women who already delivered spontaneously will profit most from a TOLA2C instead of an ERCS.

## Data Availability

The raw data are only available for analysis purposes and only to members of our research group. As the original data are hospital patient records, we do not have permission to share them.

## Abbreviations

TOLA2C: Trial of labour after two caesarean sections
ERCS: Elective repeat caesarean section
VBAC-2: Vaginal birth after two caesarean sections
C-sections: Caesarean sections
NICU: Neonatal intensive care unit

## References

[1] World Health Organization WHO. Caesarean section rates continue to rise, amid growing inequalities in access. 10.06.2024. https://www.who.int/news/item/16-06-2021-caesarean-section-rates-continue-to-rise-amid-growing-inequalities-in-access. Accessed 16 Jul 2024.

[2] Angolile CM, Max BL, Mushemba J, Mashauri HL. Global increased cesarean section rates and public health implications: a call to action. Health Sci Rep. 2023;6:e1274. 10.1002/hsr2.1274. 37216058 10.1002/hsr2.1274 PMC10196217

[3] Yaman Tunc S, Agacayak E, Sak S, Basaranoglu S, Goruk NY, Turgut A, et al. Multiple repeat caesarean deliveries: do they increase maternal and neonatal morbidity? J Matern Fetal Neonatal Med. 2017;30:739–44. 10.1080/14767058.2016.1183638. 27125601 10.1080/14767058.2016.1183638

[4] Marshall NE, Fu R, Guise J-M. Impact of multiple cesarean deliveries on maternal morbidity: a systematic review. Am J Obstet Gynecol. 2011;205:e2621–8. 10.1016/j.ajog.2011.06.035. 10.1016/j.ajog.2011.06.035 22071057

[5] Słabuszewska-Jóźwiak A, Szymański JK, Ciebiera M, Sarecka-Hujar B, Jakiel G. Pediatrics consequences of caesarean section-A systematic review and meta-analysis. Int J Environ Res Public Health. 2020. 10.3390/ijerph17218031. 33142727 10.3390/ijerph17218031 PMC7662709

[6] Gillessen A. Was kostet uns der Kaiserschnitt? Eine Analyse zur mittelfristigen Kostenbewertung und möglichen volkswirtschaftlichen Auswirkung des gesellschaftlichen Trends einer steigenden Schnittentbindungsrate im Rahmen einer Sekundärdatenanalyse [Masterthesis]. Elmshorn: NORDAKADEMIE gemeinnützige AG private Hochschule der Wirtschaft; 2015.

[7] World Health Organization WHO. WHO statement on caesarean section rates: Every effort should be made to provide caesarean sections to women in need, rather than striving to achieve a specific rate. 2015. https://iris.who.int/bitstream/handle/10665/161442/WHO_RHR_15.02_eng.pdf. Accessed 15 Jun 2024.

[8] ACOG Practice Bulletin No. 205 Summary: Vaginal birth after cesarean delivery. 2019;133:393–5. 10.1097/AOG.0000000000003079 10.1097/AOG.0000000000003079 30681536

[9] van Dillen J, Zwart JJ, Schutte J, Bloemenkamp KWM, van Roosmalen J. Severe acute maternal morbidity and mode of delivery in the Netherlands. Acta Obstet Gynecol Scand. 2010;89:1460–5. 10.3109/00016349.2010.519018. 20955100 10.3109/00016349.2010.519018

[10] Deshmukh U, Denoble AE, Son M. Trial of labor after cesarean, vaginal birth after cesarean, and the risk of uterine rupture: an expert review. Am J Obstet Gynecol. 2024;230:S783–803. 10.1016/j.ajog.2022.10.030. 38462257 10.1016/j.ajog.2022.10.030

[11] Turner MJ. Delivery after a previous cesarean section reviewed. Int J Gynaecol Obstet. 2023;163:757–62. 10.1002/ijgo.14854. 37194553 10.1002/ijgo.14854

[12] Macones GA, Cahill A, Pare E, Stamilio DM, Ratcliffe S, Stevens E et al. Obstetric outcomes in women with two prior cesarean deliveries: is vaginal birth after cesarean delivery a viable option? Am J Obstet Gynecol. 2005;192:1223-8; discussion 1228-9. 10.1016/j.ajog.2004.12.082 10.1016/j.ajog.2004.12.082 15846208

[13] Tahseen S, Griffiths M. Vaginal birth after two caesarean sections (VBAC-2)-a systematic review with meta-analysis of success rate and adverse outcomes of VBAC-2 versus VBAC-1 and repeat (third) caesarean sections. BJOG. 2010;117:5–19. 10.1111/j.1471-0528.2009.02351.x. 19781046 10.1111/j.1471-0528.2009.02351.x

[14] Sargent JA, Savitsky LM, Dissanayake MV, Lo JO, Cheng YW, Caughey AB. Gestational weight gain during pregnancy as an important factor influencing a successful trial of Labor following two previous cesareans. Am J Perinatol. 2019;36:588–93. 10.1055/s-0038-1670679. 30231273 10.1055/s-0038-1670679 PMC7138436

[15] Horgan R, Hossain S, Fulginiti A, Patras A, Massaro R, Abuhamad AZ, et al. Trial of labor after two cesarean sections: a retrospective case-control study. J Obstet Gynaecol Res. 2022;48:2528–33. 10.1111/jog.15351. 35793784 10.1111/jog.15351 PMC9796916

[16] Maroyi R, Nyakio O, Buhendwa C, Mukanga O, Kalunga K, Kanyinda K, et al. Experience on trial of labor and vaginal delivery after two previous cesarean sections: a cohort study from a limited-resource setting. Int J Gynaecol Obstet. 2023;162:266–72. 10.1002/ijgo.14665. 36708063 10.1002/ijgo.14665

[17] de Leo R, La Gamba DA, Manzoni P, de Lorenzi R, Torresan S, Franchi M, Uccella S. Vaginal birth after two previous cesarean sections versus elective repeated cesarean: a retrospective study. Am J Perinatol. 2020;37:S84–8. 10.1055/s-0040-1714344. 32898889 10.1055/s-0040-1714344

[18] Rotem R, Hirsch A, Sela HY, Samueloff A, Grisaru-Granovsky S, Rottenstreich M. Maternal and neonatal outcomes following trial of labor after two previous cesareans: a retrospective cohort study. Reprod Sci. 2021;28:1092–100. 10.1007/s43032-020-00378-1. 33185861 10.1007/s43032-020-00378-1

[19] World Medical Association Declaration of Helsinki. Ethical principles for medical research involving human subjects. JAMA. 2013;310:2191–4. 10.1001/jama.2013.281053. 24141714 10.1001/jama.2013.281053

[20] von Elm E, Altman DG, Egger M, Pocock SJ, Gøtzsche PC, Vandenbroucke JP. The strengthening the reporting of observational studies in epidemiology (STROBE) statement: guidelines for reporting observational studies. Ann Intern Med. 2007;147:573–7. 10.7326/0003-4819-147-8-200710160-00010. 17938396 10.7326/0003-4819-147-8-200710160-00010

[21] Landon MB, Spong CY, Thom E, Hauth JC, Bloom SL, Varner MW, et al. Risk of uterine rupture with a trial of labor in women with multiple and single prior cesarean delivery. Obstet Gynecol. 2006;108:12–20. 10.1097/01.AOG.0000224694.32531.f3. 16816050 10.1097/01.AOG.0000224694.32531.f3

[22] Davidson C, Bellows P, Shah U, Hawley L, Drexler K, Gandhi M, et al. Outcomes associated with trial of labor after cesarean in women with one versus two prior cesarean deliveries after a change in clinical practice guidelines in an academic hospital. J Matern Fetal Neonatal Med. 2020;33:1499–504. 10.1080/14767058.2018.1520831. 30185092 10.1080/14767058.2018.1520831

